# A Case Study on the Integrated Healthcare Delivery System in Xuhui District, Shanghai Best Practices and Implementation

**DOI:** 10.5334/ijic.9812

**Published:** 2026-07-02

**Authors:** Yanjun Huang, Gan Wang, Rong Chen, Biao Wang, Xinyu Li, Qiong Zhu, Zhijie Yu

**Affiliations:** 1 Kangjian Community Health Service Center, Xuhui District, Shanghai, China; 2 Shanghai Hospital Development Center, Shanghai, China; 3 School of Public Health, Fudan University, Shanghai, China

**Keywords:** integrated eldercare, Six-Bed Integration model, care coordination, resource allocation, referral mechanism, digital divide, community-based elderly care

## Abstract

**Background:**

The “Six-Bed Integration” model in Kangjian Subdistrict, Shanghai, represents an innovative approach to integrated eldercare, combining three healthcare beds (family medical beds, rehabilitation/palliative care beds, and hospital beds) with three eldercare beds (home care, day care, and institutional care). This study explores the implementation challenges and systemic tensions within this model.

**Methods:**

Using purposive sampling, professionals (N = 15) involved in the Kangjian integrated care system were interviewed through semi-structured interviews. Data were analyzed via Colaizzi’s phenomenological approach to identify key themes.

**Results:**

The study identified three key challenges in the integrated care model: (1) Structural barriers including Medicare restrictions, bed shortages, and unclear admission criteria hindered service integration; (2) Ineffective outreach to homebound seniors and digital disparities limited service accessibility and understanding; (3) Unclear stakeholder responsibilities caused referral disputes, with nursing homes rejecting high-risk cases and discharge-bed availability mismatches worsening conflicts. These findings reveal systemic gaps in resource allocation, communication, and coordination within the integrated care system.

**Conclusion:**

The “Six-Bed Integration” model demonstrates potential in bridging medical and social care but faces challenges in resource allocation, communication, and inter-institutional accountability. The study implies that optimizing integrated care delivery requires not only frontline training in coordination skills but also policy support to create synergistic mechanisms for resource sharing and accountability. Policy interventions must address Medicare constraints, standardize referral protocols, and enhance cross-sector collaboration to optimize integrated eldercare delivery.

## Introduction

### Research Background and Policy Environment

According to the World Health Organization (WHO), the global population is aging at a significantly accelerated pace compared to historical trends. The demographic cohort aged 60 years and above is projected to escalate from 1 billion in 2020 to 1.4 billion. By 2050, this geriatric population segment will have doubled, reaching 2.1 billion individuals worldwide [1]. China possesses the world’s largest elderly population, with Shanghai emerging as a paradigmatic case of pronounced population aging [2]. According to the latest statistical data of Shanghai Civil Affairs Bureau, by the end of 2023, the population aged 60 and above accounted for 37.4% of Shanghai’s total resident population. Numerous non-communicable chronic diseases, particularly multimorbidity patterns, exhibit elevated prevalence rates among its geriatric demographic, notably manifesting in cognitive impairment, diabetes mellitus, hypertension, Parkinson’s disease, and osteoporosis [3]. For instance, a nationwide study of Chinese adults aged 60 years and above revealed a cognitive impairment-diabetes gradient, with prevalence rates escalating from 26.0% in mild cognitive impairment to 36.9% in dementia cases [4].

With advancing age, the progressive decline in physiological capacity elevates susceptibility to a spectrum of age-related pathologies. The Integrated Care for Older People (ICOPE), proposed by WHO, offers a comprehensive, person-centered framework to maintain intrinsic and functional capacity through coordinated health and social services [5]. It emphasizes evidence-based, multidimensional strategies combining community interventions, cross-sectoral coordination, and personalized integration to support autonomy in older adults [6]. Unlike conventional fragmented care in China, integrated care connects medical, social, and eldercare services via multidisciplinary teams and standardized assessments, proactively addressing capacity decline and preventing avoidable hospitalizations, while reducing duplication and resource inefficiency [7].

Since 2015, the Chinese government has promulgated a series of pivotal policy documents at the national level, notably including the *‘Guidelines on Promoting Contracted Family Doctor Services’*(National Health Reform Office [2016] No. 1), the *‘Guiding Opinions on Promoting the Construction and Development of Medical Consortia’* (State Council General Office [2017] No. 32), the ‘*Administrative Measures for Medical Consortia (Trial Implementation)’* (National Health Commission Office [2020] No. 13) and the *‘Guiding opinions on comprehensively promoting the construction of compact county medical and health community’*(Department of Primary Health [2023] No.41) [8,9,10,11]. These initiatives have institutionalized a multi-tiered integrated care framework encompassing: (i) grid-based urban medical clusters and county-level healthcare networks, (ii) specialized alliances for critical diseases and scarce resources, and (iii) personalized family physician services. These align with Healthy China 2030 and the 14th Five-Year Plan’s mandates for integrated healthcare-social support systems through structural innovation.

### Development and Challenges of the Practice of Combining Medical Treatment with Nursing Care

Numerous countries have initiated research on implementation frameworks and government-led plans for integrated care in the early stages. Japan’s 2012 Long-Term Care Insurance Act introduced the Community-Based Integrated Care System, scheduled for full implementation by 2025. This model integrates acute medical and long-term care services under insurance coverage to deliver continuous community-based support for older adults with chronic conditions or disabilities, while containing social security costs. It emphasizes tri-level integration (system, organization, clinical) through multidisciplinary teams including public health nurses and care managers to provide personalized, continuous care [12]. In 2015, NHS England launched the Vanguard Integrated Care Programme, later incorporated into the 2019 NHS Long Term Plan. Covering 9% of England’s population, this initiative coordinates health, social care and rehabilitation services through cross-organizational collaboration, particularly for community-based patients. Results showed a 6% slower growth rate in emergency admissions across 23 pilot sites compared to controls, demonstrating effective hospital demand management [13]. The INSPIRE study in Toulouse, France demonstrated that implementing WHO’s ICOPE model that focuses on five intrinsic capacity (IC) domains (mobility, cognition, nutrition and vitality, psychology, sensory) effectively replaces disease-centered care. Key findings revealed significant associations between biological aging markers and IC decline, supporting biological interventions to preserve function [14]. An integrated care programme that was mandated by the UK’s General Medical Services contract uses a standardized electronic assessment, the Luton Framework for Frailty (LFF), to identify frailty in older adults and automatically link them to tailored interventions [15,16].

The evolution of integrated care research in China has progressed through three distinct developmental phases: the incipient stage (2000–2007), characterized by theoretical germination and conceptual exploration; the exploratory stage (2008–2015), marked by systematic investigations into context-appropriate integrated models and multi-stakeholder interest coordination mechanisms; and the maturation stage (2016–2020), during which researchers not only delineated implementation challenges but also advanced pragmatic solutions, including shared governance frameworks and person-centered service delivery models to address systemic fragmentation [17]. Multiple studies have pointed out that there is a lack of systematic integration among medical, elderly care, psychological, and social services in China [18,19,20]. Information silos are widespread, leading to repeated medical visits, resource wastage, and fragmented care for older adults. Care workers and primary healthcare providers often lack sufficient capabilities in managing multimorbidity, cognitive impairment, and providing psychological support, which negatively impacts service quality. The concept of “integrated care” is subject to varying interpretations across sectors, and the absence of standardized training frameworks has contributed to a significant shortage of adequately trained professionals in the field [18,21].

Assessment tools and service models also lack localization and standardization. For example, while the ICOPES-TW screening tool demonstrates feasibility, it remains in the early validation phase and has not yet been widely adopted in community or institutional care settings. The absence of unified evaluation indicators and service processes leads to inconsistencies in care quality and hinders scalability [21,22]. The LFF assessment tool has only been studied in the context of implementation in the UK, and it has not yet been introduced to China [23,24]. In government procurement of services, resource allocation still prioritizes cost control over outcomes such as user satisfaction or care effectiveness, failing to reflect a people-centered investment approach [22]. Additionally, older adults and their families generally have limited awareness of integrated care services. Some continue to place greater trust in traditional medical or home-based care models and express uncertainty or reluctance toward multi-sectoral, collaborative approaches [18]. As a result, the common challenges facing the development of integrated care in China include: service fragmentation, insufficient primary care capacity, lack of standardized tools and models, unclear performance-based funding mechanisms, and low public acceptance.

### Innovation of “Six-Bed Integration” Model in Xuhui District

As one of Shanghai’s top five most aged central districts, Xuhui District recorded a 36.45% proportion of residents aged 60 and above in 2022. The “9073 model” is an eldercare framework where 90% of seniors receive home-based care, 7% utilize community services, and 3% reside in institutional facilities in Shanghai [25]. Since 2024, Kangjian Subdistrict in Xuhui District has piloted an innovative “Six-Bed integration” model, which reconfigures the traditional “9073” eldercare framework by integrating three types of medical beds (home-based, rehabilitation/palliative, and hospital beds) with three types of care beds (home care, day care, and institutional care). This model enables dynamic allocation of service resources and addresses the fragmentation of care settings within the conventional “9073” system [26]. Anchored by the Kangxin Integrated Eldercare Center and the community health service center, the model establishes cross-setting service pathways: home-based older adults receive medical support via home beds; day care centers coordinate with rehabilitation beds for functional training; and institutional care is linked with palliative beds to form a closed-loop end-of-life care system.

The integration mechanism operates on three levels: (1) tiered response to needs: priority support is provided to high-risk older adults living alone (e.g., 2,260 targeted individuals) through coordinated home care and home medical beds; (2) intelligent resource allocation: digital platforms enable streamlined referrals between medical and institutional beds; and (3) progressive service continuity: day care users can transition to medical or rehabilitation beds as care needs escalate.

This model is led by the Xuhui District Civil Affairs Bureau, in collaboration with the District Health Commission and the Medical Insurance Bureau, forming a tripartite coordination mechanism characterized by “civil affairs leadership, health commission collaboration, and medical insurance support.” The specific division of responsibilities is as follows: the civil affairs department is responsible for integrating elderly care bed resources and establishing service standards; the health department oversees medical services for home-based hospital beds, rehabilitation beds, and palliative care beds; and the medical insurance department is tasked with exploring payment methods aligned with integrated care [27].

### Research Questions

Given the innovative yet under-examined nature of the “Six-Bed Integration” model, this study addresses the following core question: How does the “Six-Bed Integration” model operate in practice, and what are the key barriers to its successful implementation? As a pioneering initiative in one of China’s most aged urban districts, this case study seeks to provide critical insights into the real-world integration of healthcare and eldercare services, with implications for policy and practice both nationally and internationally.

## Ethical Approval

This study was approved by the Shanghai Ethics Committee for Clinical Research (Approval No.: SECCR/2024-231-01) and strictly adhered to the Chinese Ethical Guidelines for Social Science Research. SECCR is a centralized, independent ethical review platform established specifically for clinical studies at the municipal level. Its review decisions are widely recognized by numerous medical institutions in Shanghai and even across the country. All participants provided signed informed consent, which clearly outlined the research purpose, anonymization of data, and the right to withdraw voluntarily. Respondents were referred to by coded identifiers (N1–N15), and sensitive information was desensitized.

## Methods

### Research Subjects

Purposive sampling was employed to select staff members: (1) at least half a year of involvement in the multi-bed integrated care program in Kangjian Subdistrict since September 2024, and (2) at least two years of work experience in medical or eldercare services. Exclusion criteria: (1) non-participation in the multi-bed integrated care program, and (2) unwillingness to provide informed consent for the interview. Based on these criteria, 15 staff members were selected as respondents (coded N1–N15). Sociodemographic characteristics of participants are presented in Table 1.

**Table 1:** Sociodemographic Characteristics of Participants (N = 15).


CHARACTERISTIC	CATEGORY	COUNT (n)	PERCENTAGE (%)

Gender	Female	14	93.3

	Male	1	6.7

Age (years)	20–29	2	13.3

	30–39	5	33.3

	40–49	8	53.3

District	Xuhui District	15	100.0

Institution	Kangjian CHSC	12	80.0

	Kangxin Elderly Care	2	13.3

	Zhongque Elderly Care	1	6.7

Professional Title	Senior Title (Associate Chief Physician/Senior Nurse)	4	26.7

	Intermediate Title (Attending Physician)	3	20.0

	Junior Title (Junior Staff)	5	33.3

	Other (Social Worker, etc.)	3	20.0

Education	Bachelor’s Degree	14	93.3

	Master’s Degree	1	6.7

Years of Experience	≤5	4	26.7

	6–15	4	26.7

	16–25	6	40.0

	≥26	1	6.7


*Note:* CHSC = Community Health Service Center. Percentages are calculated based on the total sample size (N = 15) and may not sum to 100% due to rounding.

### Interview Guide Development

Semi-structured interviews were conducted for data collection. An interview guide was drafted based on research objectives and a review of relevant domestic and international literature [28,29,30]. In particular, the framework proposed by Zhang et al. [30], which delineates factors influencing experiences, including environmental design, individual perceptions, and interpersonal interactions, served as a basis for formulating questions on service coordination and continuity. Additionally, evidence from studies addressing implementation challenges, such as Li et al. [28,29], and Arku D et al. [28,29], on workforce capacity and multidisciplinary collaboration, provided critical insights for shaping questions related to current service outcomes and future development directions. Input from experienced healthcare professionals and long-term eldercare service providers was incorporated. Two staff members were pre-interviewed to refine the content before finalizing the guide. Key questions included:

- How does Kangjian Subdistrict’s integrated healthcare and eldercare system coordinate community, home-based, and institutional services?
- Within the six-bed integration system, how is effective referral and continuity ensured across different service beds?
- What are the current outcomes of integrated healthcare and eldercare services? Are there evaluation data or case studies to share?
- What are Kangjian Subdistrict’s future plans and development directions for integrated healthcare and eldercare?
- How can service coverage and quality be further improved to better meet older adults’ needs?

### Data Collection Methods

One-on-one semi-structured interviews balanced preset research frameworks with participant narrative flexibility. After obtaining consent, interviews were conducted offline face-to-face at agreed times/locations. Researchers explained the study’s purpose, content, and significance beforehand. Each interview averaged 45 minutes, with audio recording (participant-approved) and non-verbal cues documented. All recordings were kept confidential. Fifteen interviews were transcribed verbatim. Colaizzi’s systematically extracts meanings from lived experiences, clusters themes on structural and coordination challenges, and validates findings with participants, ensuring rigor and credibility in exploring the complex dynamics of the Six-Bed Integration model [31,32,33]. This study employs Colaizzi’s seven-step phenomenological method to systematically analyze the interview data. Following Colaizzi’s seven-step method, transcripts were read repeatedly to grasp overall meaning. Significant statements were extracted and meanings formulated. These were organized into theme clusters reflecting barriers and coordination challenges. The findings were integrated into an exhaustive description, which was then condensed into essential structures. Finally, results were returned to participants for validation, ensuring accuracy and credibility.

## Units of Analysis

### Kangxin Integrated Elderly Care Service Center (Xuhui District, Shanghai)

Affiliated with Kangjian Subdistrict, Kangxin Center launched a pilot in September 2024 with support from the District Civil Affairs Bureau and Health Commission. It integrates medical, rehabilitation, and eldercare resources for community and institutional settings, tailoring services to diverse needs. As the hub of Kangjian’s integrated healthcare-eldercare system, it coordinates all local eldercare and medical resources, providing outreach, education, counseling, referrals, and daycare—forming the core of the six-bed integration.

### Kangjian Community Health Service Center (Xuhui District, Shanghai)

**Figure 1:**
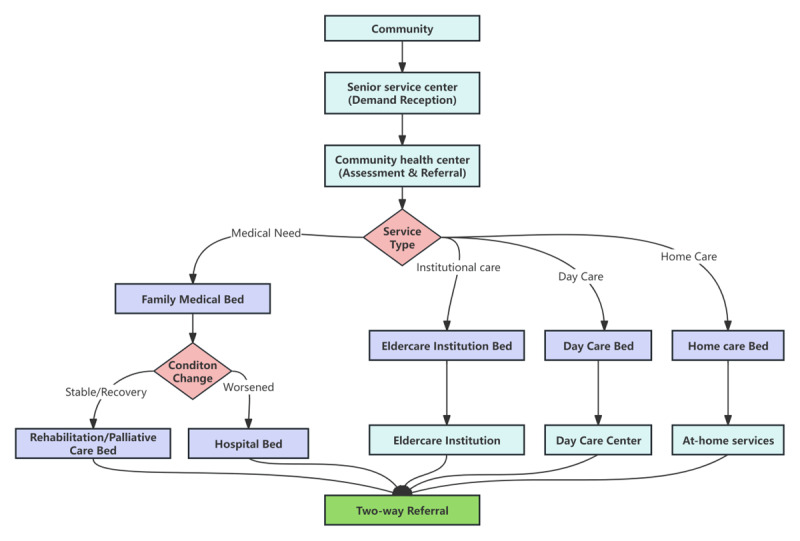
Kangjian Subdistrict’s Six-Bed Integrated Care Network (Xuhui District, Shanghai). (Eldercare services include: Institutional beds (Xuhui Social Welfare, Xinle Elderly Care, Kangjian Nursing Home, Changqingfang & Shouchangfang Care Centers); Home care beds (Xuhui No.3 Senior Welfare, Shouchangfang Center); Day care at Kangxin Senior Service Center; Medical services through Kangjian CHC (family/rehab/palliative beds) and Shanghai 8th People’s Hospital; Dingxiangyuan Senior Center currently vacant. The cross-institution referral process is coordinated by the Kangjian Subdistrict Integrated Elderly Care Service Center as the central hub. Through a joint assessment team (including family physicians, heads of elderly care institutions, and social workers), it conducts needs assessments and bed matching to achieve closed-loop management of “assessment-referral-admission-feedback.”).

As Shanghai’s first integrated healthcare-eldercare pilot subdistrict, Kangjian established its system and six-bed integration model to deepen service integration. The center manages three beds: eldercare beds, home-based hospital beds, and rehabilitation/palliative care beds (primary hospital treatment beds). Through Kangxin Center, families’ needs and seniors’ conditions are assessed for eligibility. Recognized as a national model, Kangjian’s “six-bed integration” is illustrated in the two-way referral pathway (Figure 1). It links family medical, rehabilitation/palliative, hospital, eldercare institution, day care, and home care beds, bridging institutional, community, and home-based services. The two-way referral operates across all bed types, enabling older adults to shift flexibly between care settings as conditions change, ensuring continuity, reducing fragmentation, and achieving seamless upward and downward transfers.

### Resident Health Profile

The center has established a comprehensive public health system through integrated medical-education-research collaboration and community-hospital partnerships, emphasizing prevention and lifelong health management. Supported by electronic health records and 5G technology, it delivers end-to-end services from profiling and screening to treatment and education. Coverage has reached 92.35% of the subdistrict’s 96,000 residents, with full registration for 39,591 contracted residents and dynamic updates for 82.15%. Services for priority groups are notable: 21,389 pediatric visits (99.3% coverage), early prenatal registration for 296 women, and targeted support for 2,512 octogenarians (including 13 centenarians) and 151 bereaved families. Chronic disease management has achieved standardized control rates of 88.49% for hypertension (13,720 cases), 90.46% for diabetes (4,531 cases), and 99.04% for mental health conditions. These outcomes highlight the center’s capacity to deliver high-quality, precision public health services, advancing its vision of preventive, full-cycle health governance.

Collectively, the development of Kangxin and Kangjian centers, together with the Resident Health Profile, consolidates rich databases, digital management, and skilled nursing teams, providing essential infrastructural and professional support that underpins the effective piloting and advancement of the Six-Bed Integration model.

## Results

### Case Study Analysis

#### Participant Characteristics by Service Type

The Patient Care Risk Assessment Form covers nine items: falls, falling from bed, choking, accidental ingestion of medications/food, harm to self or others, burns, wandering/getting lost, pressure ulcers, and recreational activity-related accidents. Participant characteristics by service type are detailed in Table 2.

**Table 2:** Participant Characteristics by Service Type.


NAME	SERVICE TYPE	AGE	GENDER	OCCUPATION	CHRONIC CONDITIONS	CARE RISKS*	PATIENT HEALTH STATUS AND CARE SETTING TRANSITIONS

Patient A	Community-Based Patient Transfer to Daycare Respite Service	73	Male	Secondary school teacher	Diabetes	None	A 72-year-old male with long-term diabetes mellitus residing in Community A was referred to Kangxin Integrated Elderly Care Service Center’s daycare program. Previously institutionalized at a long-term care facility, the patient (with a history of diabetic toe amputations and mobility impairment) was transitioned to daycare services on December 6th following hospital discharge. His spouse reported difficulties in providing adequate home care due to age-related limitations and frequent absences, particularly concerning fall risks and environmental hazards. The daycare program provided professional supervision, cognitive-physical activities, and social engagement opportunities.
	
Patient B		88	Male	Military veteran	Hypertension	None	An elderly couple from Community B demonstrated significant lifestyle improvement after enrolling in Kangxin’s services in July 2024. Prior to engagement, their daily activities were limited to essential shopping and walking. Post-enrollment, they regularly participated in structured activities (handicrafts, calligraphy, reading, and film therapy), which enhanced social connectivity and mental stimulation, culminating in their formal appreciation through commendation letters.
	
Patient C		86	Female	Warehouse administrator	Hypertension	Moderate cognitive impairment	
	
Patient D		81	Male	Design institute engineer	Hypertension	None	A married couple faced institutional care barriers due to geographical separation, with the wife previously residing in a nursing home in Qingpu District. After learning about Kangjian Subdistrict’s six-bed integration system in September 2024, they opted for daycare services following comprehensive geriatric assessment (including physical function, self-care capacity, and cognitive risk evaluation). The wife now receives daily professional care with non-pharmacological cognitive interventions while maintaining nighttime cohabitation.
	
Patient E		77	Female	Food factory clerk	Diabetes, Parkinson’s disease	Mild cognitive impairment risk	

Patient F	Transfer from Elderly Care Service Center to Senior Care Facility Bed	77	Female	University professor	Spinal injury, Scoliosis, Osteoporosis	None	A homebound, medically fragile solo ager without guardians applied for palliative care admission after learning about the program through official media. Following a February 12 home assessment and care coordination, the patient was admitted to Xinle Nursing Home for enhanced medical accessibility.

Patient G	Transfer from Rehabilitation Bed to Senior Care Facility Bed	76	Female	Unemployed	Cerebral infarction	None	A bedbound, nonverbal patient (Long-term Care Insurance Level 6) was transferred from Kangjian Community Health Center to Xinle Nursing Home on October 11, 2024, requiring complete assistance with activities of daily living (ADLs).
	
Patient H		85	Female	Unemployed	Lumbar fracture	None	An alert, verbally communicative patient (Long-term Care Insurance Level 3) with preserved ADL capacity was transitioned to Xinle Nursing Home in March 2025.

Patient I	Transfer from Home-Based Hospital Bed to Rehabilitation Care Bed	92	Female	Professional technician	Diabetes, Hypertension	High pressure ulcer risk	Two patients were transferred from home-based hospital beds to Kangjian’s rehabilitation units in March 2025: one in critical condition with somnolence (Case 8, March 3), and another stabilized with partial ADL independence (Case 9, March 12).
	
Patient J		93	Male	Unemployed	Diabetes, Hypertension	None	


*Note:* * The Patient Care Risk Assessment Form covers nine items: falls, falling from bed, choking, accidental ingestion of medications/food, harm to self or others, burns, wandering/getting lost, pressure ulcers, and recreational activity-related accidents.

#### Transfer from Community–Based Patient to Daycare Respite Service

Case 1: Applicants consulted the senior care center and, after a successful assessment, enrolled eligible older adults with disabilities in daycare respite services (Patients A–E). These community-based services provide daytime care to alleviate family burdens and enhance seniors’ quality of life.

#### Transfer from Elderly Care Service Center to Senior Care Facility Bed

Case 2: A community resident requiring senior care facility admission was assessed on-site by a team coordinated through the Elderly Care Service Center. The team, including a family doctor and the facility head, evaluated the patient’s medical condition, daily activity ability, and family support. The patient was admitted after meeting the criteria (Patient J).

#### Transfer from Rehabilitation Bed to Senior Care Facility Bed

Case 3: A patient stabilized in a community center rehabilitation bed was admitted to the on-site Xinle Nursing Home after a joint assessment by their attending physician and the nursing home head confirmed they met the criteria (Patients F–G).

#### Transfer from Home–Based Hospital Bed to Rehabilitation Care Bed

Case 4: A home-based patient whose condition worsened was admitted to the center’s rehabilitation ward after a joint assessment by the family doctor and a rehabilitation physician confirmed eligibility (Patients H–I). The assessment included medical condition and Karnofsky Performance Status (KPS).

### Key Challenges

#### Synergistic Effects and Structural Contradictions in Resource Integration

**Figure 2:**
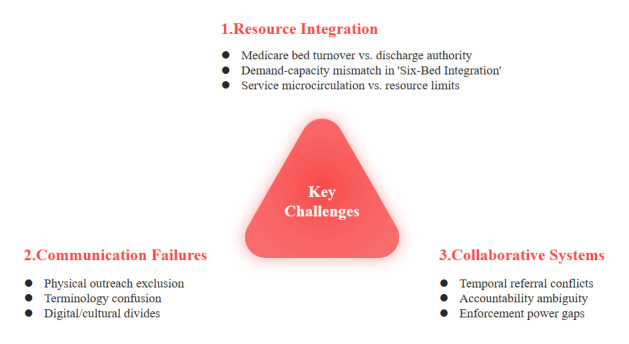
Key Challenges in the Kangjian Subdistrict’s Six-Bed Integrated Care Network.

The practice of integrated eldercare and healthcare demonstrates multi-level spatial restructuring characteristics. Community health center staff explicitly stated: “*The Medicare restrictions on bed turnover are the fundamental contradiction—we have no authority to discharge patients, but Medicare strictly regulates us*” (N1). This institutional tension is particularly evident in the “Six-Bed Integration” model. Although the parallel deployment of home medical beds and home care beds theoretically achieves “*seamless medical-eldercare integration*” (N3: “family physicians provide regular home visits”), rehabilitation bed operators lamented: “*Demand overwhelms capacity. Referred seniors without guardians don’t meet admission criteria, exposing us to malpractice risks*” (N13). Notably, the embeddedness of family doctor teams in nursing homes was termed “*service microcirculation*” (N2), yet this innovation faces resource constraints: “*As the city’s pilot site, our three medical beds (rehabilitation, hospice, and home-care beds) are perpetually insufficient*” (N1). Key challenges in the Kangjian Subdistrict’s Six-Bed Integrated Care Network are illustrated in Figure 2.

The involvement of social forces reveals boundary disputes in public service delivery. While third-party organizations alleviate “*bed shortages that may trigger disputes*” (N4), nursing home administrators highlight deeper systemic gaps: “*We lack clarity on follow-up services from senior care centers, making referrals risky*” (N13). Such distrust reflects institutional ambiguities between market-based supplementation and public accountability.

#### Phenomenological Deconstruction of Communication Failures

Traditional outreach suffers from physical limitations that systematically exclude key populations. “*We distribute leaflets and host events, but bedridden elders never see them*” (N4). This “*presence barrier*” distorts service coverage rates. More critically, cognitive translation failures occur—one family member noted: “*The brochure’s ‘daycare beds’ confused my mother; she thought they were hospital beds and refused to apply*” (N5).

The digital divide exacerbates information asymmetry. Despite hotline services, staff admitted: “*Adult children should mediate communication, yet we haven’t even established WeChat groups*” (N2). Cultural disparities further fracture messaging—”*Elders interpret our ‘Kang Xiao Yang’ mascot differently: some find it endearing, others childish*” (N5). This fragmented outreach stems from poor community synergy: “*Neighborhood committees, property managers, and our teams work in silos without shared needs assessments*” (N4).

#### Tensions and Boundary Disputes in Collaborative Systems

Temporal conflicts over scarce resources peak during referrals. Medical bed managers emphasized: “*Hospital stays have strict limits, but families beg, ‘Wait a few more days for nursing home vacancies’*” (N4), forcing community health centers into dilemmas: “*Discharge delays block new patients, and we’re blamed for unethical practices*” (N1).

Ambiguous accountability fuels interagency friction. Senior service centers criticized: “*Hospitals push discharge assessments to us, but nursing homes reject ‘high-risk’ referrals*” (N1), while care facilities retorted: “*Medicare audits hospitals, not us—why should we bear their risks?*” (N13). This institutional deadlock persists—”*Street offices mandate our involvement, but laws grant no enforcement power*” (N1).

Across the presented referral cases, several commonalities and differences emerge. On the one hand, joint assessments facilitated smoother transitions by reducing family burden and enabling timely access to community or institutional resources. On the other hand, disparities were evident in admission criteria, service availability, and the degree of coordination among providers, particularly for high-risk or unaccompanied elderly. Collectively, these cases reveal systemic challenges inherent to the “Six-Bed Integration” model, including misaligned admission protocols, limited bed supply, and inconsistent referral pathways. The root causes of these challenges extend beyond individual institutions. Policy frameworks, especially medical insurance payment rules, incentivize rapid hospital discharge but lack flexible mechanisms to support continuity of care. Departmental silos between health, civil affairs, and social service agencies exacerbate coordination gaps and accountability disputes. At the industry level, persistent shortages of trained geriatric care professionals limit service capacity, while uneven levels of digitalization hinder real-time information sharing across sectors. These structural constraints underscore that the “Six-Bed Integration” model, while innovative, must be reinforced by systemic reforms in financing, governance, workforce development, and digital infrastructure to achieve sustainable integrated care.

## Discussion

### Institutional Barriers and Structural Contradictions in Resource Integration

Institutional barriers in resource integration arise from disconnects between policy frameworks and service realities. Rigid healthcare insurance mechanisms conflict with the extended care needs of frail elderly populations. For example, risk-averse admission protocols systematically exclude unaccompanied seniors. These systemic failures are compounded by weak multi-sector coordination. Information gaps persist in cross-institutional referrals, and care facilities shoulder disproportionate risks because legal frameworks for shared accountability are absent. Artificial scarcities also persist. Pilot medical beds fail to meet community demand, and compulsory hospital discharge timelines clash with limited facility capacity. This produces a governance trilemma: cost containment through bureaucratic control, equitable access, and quality assurance cannot all be achieved simultaneously.

Similar challenges are seen internationally. In France, elderly care integration pilots are frequent, but they are led by siloed administrative bodies without horizontal coordination or accountability mechanisms. Local practitioners attempt “integrative piloting” to bridge systemic gaps. Yet, without national legal support, collaboration depends heavily on personal networks and individual initiative, preventing large-scale integration [34].

Structural barriers also include fragmented discharge planning by non-governmental organizations, limited facility capacity, and unclear role definitions. These factors create information discontinuities and concentrate risks on single actors, highlighting the lack of a cross-sector risk-sharing framework [35]. Ultimately, institutional paralysis stems from three unresolved tensions: mismatches between segmented administrative systems and continuous care needs, the absence of cross-sector risk mitigation mechanisms, and chronically ambiguous legislative mandates for integrated care models [36]. Although this study primarily adopts a qualitative approach, preliminary findings—supplemented by pilot data from the Kangjian Community Health Service Center in 2024 (e.g., a 21% increase in home-based hospital bed utilization and a 56% success rate in referrals to elderly care institutions)—suggest the model’s potential to enhance service accessibility and resource utilization efficiency. Future research should incorporate quantitative indicators (such as bed turnover rates, patient satisfaction, and changes in medical costs) to further validate its incremental value.

### Service Accessibility and Fracture of Information Dissemination

The accessibility of services is structurally undermined by systemic discontinuities in information dissemination, where critical care resources remain operationally disconnected from target populations due to fragmented communication channels and uncoordinated outreach mechanisms. This rupture manifests through two interdependent dimensions: physical information barriers that exclude bedbound elders from service awareness, and cognitive translation failures wherein words like ‘daycare beds’ is misinterpreted by intended beneficiaries. The disjunction is further exacerbated when digital outreach platforms assume family mediation roles without establishing necessary intergenerational communication infrastructures, sunch as unestablished WeChat groups, while cultural mismatches in health messaging, such as divisive perceptions of “Kang Xiao Yang” mascots, reveal unaddressed sociolinguistic variances across communities. Research indicates that virtual healthcare visits demand a high level of technological proficiency, yet many socially vulnerable seniors face significant barriers related to limited access to technology and inadequate training—such as lacking assistance in operating devices or being unable to join WeChat groups [37].

Language barriers and low health literacy are found to be closely associated with poor self-rated health. Even when linguistically concordant healthcare services are provided, issues of cultural mismatch remain insufficiently addressed [37]. Ultimately, this represents not merely an information gap but a fundamental collapse in the care continuum’s communicative architecture, where unshared needs inventories between neighborhood committees, property managers, and service providers institutionalize exclusion through bureaucratic silos rather than technical limitations.

### Referral Mechanism and Reconstruction of Power and Responsibility System

The referral mechanisms and accountability systems require fundamental restructuring. This is evident in the operational tensions within the “Six-Bed Integration” model in Xuhui District. Systemic dysfunction arises when hospital discharge protocols, such as medical insurance restrictions on bed turnover, conflict with the risk-averse practices of nursing institutions. These facilities often refuse admission to unaccompanied elderly patients. This misalignment produces a contradictory situation. Hospitals are penalized for retaining patients beyond the permitted period. Downstream care providers reject medically stable elderly individuals because of liability concerns. Community health centers lack binding authority to enforce transitional care. The result is an accountability vacuum that shifts the burden to family caregivers and perpetuates long-term bed-blocking crises.

Three dimensions of governance failure are exposed. First, there is a mismatch between discharge schedules and placement delays (time). Second, responsibility is distributed asymmetrically across institutions (risk). Third, enforcement authority remains unclear (jurisdiction). Social disadvantages, such as living alone, further increase the likelihood of nursing home admission after discharge. This reveals a weak integration of social support and medical assessment, which shifts responsibility from hospitals to care institutions and worsens bed-blocking problems [38].

More broadly, the elderly care system suffers from widespread institutional misalignments. Policies and regulations are inconsistent. Responsibility is spread across multiple actors. Collaboration between organizations is weak, and information exchange is inadequate. These mismatches reduce efficiency, obstruct coordination, and prevent shared accountability [39]. The findings echo international evidence on barriers to integrated care. Yet, they also highlight a uniquely Chinese tension between vertical administrative control, enforced through health insurance performance assessments, and the horizontal need for service integration.

### Theoretical Enlightenment and Practical Innovation

The “Six-Bed Integration” case exemplifies the distinctive challenges China faces in developing an integrated eldercare system, specifically, a threefold mismatch involving bureaucratic efficiency imperatives, deeply rooted familial care traditions, and the rapid emergence of market mechanisms. These tensions are further intensified by the demographic reality of hyper-urban aging, as illustrated by Xuhui District’s population, where 36.45% are aged 60 and above.

Although China shares certain structural challenges with international models—such as Japan’s Community-Based Integrated Care System, which seeks to balance insurance constraints with continuity of care, or England’s Vanguard Programme, which enhances cross-sector coordination and reports a 6 percent reduction in emergency admissions—the Xuhui model offers unique contrasts. While all face issues of coordination and accountability, Xuhui highlights acute pressures from rapid urban aging, uneven insurance coverage, and limited community capacity. These lessons are particularly relevant for low-resource settings, where targeted referral protocols, digital monitoring, and shared risk-pooling mechanisms can provide scalable pathways to sustainable integrated care.

China’s situation is marked by three uniquely intensified pressures. First, the policy timeline is unusually compressed, as the country attempts to address decades of aging-related challenges within a matter of years. Second, there exists a pronounced asymmetry in resource distribution, particularly between well-resourced tertiary hospitals and underdeveloped community care infrastructure. Third, the allocation of care responsibilities remains unresolved, with ongoing ambiguity over the roles of the state, market, and family caregivers.

### Policy Implications

The findings reveal structural contradictions within the integrated care system at the institutional, service, and referral levels, offering clear directions for policy development. In the short term, it is necessary to establish mandatory referral coordination mechanisms and a digital bed-monitoring platform, while piloting a “risk-pooling fund” jointly supported by medical insurance, civil affairs, and care institutions to address admission challenges for unaccompanied older adults [38,39]. In the long term, reforms should promote the integration of medical insurance and long-term care insurance funds, with capitation-based payments for integrated service packages; establish a specialized track in “Geriatric Integrated Care” within medical schools to train interdisciplinary teams; and legislate for the sharing of electronic health records alongside unified service quality evaluation standards to ensure continuity of care [40,41]. These measures aim to resolve the tension between vertical administrative control and horizontal service integration, thereby building a sustainable integrated care system.

### Research Limitations and Future Directions

This study has three main limitations. First, the sample lacked representativeness, with only 15 professionals interviewed and limited inclusion of family or elderly perspectives, weakening user-side analysis. Second, the absence of process indicators like bed turnover and referral success rates restricted precise evaluation of system efficiency. Third, A limitation of this study is the lack of methodological triangulation to further enhance the validity and reliability of the findings.

Future research may explore micro-level issues such as quantifying resource misallocation and communication failures, and system-level tensions by applying collaborative governance theory to examine interactions among healthcare, eldercare institutions, and communities. The study sample primarily consisted of participants from community health service centers (80%) and elderly care institutions (20%), with no representation from other organizations such as medical insurance departments, civil affairs management agencies, or other relevant government departments. This sample composition may limit the in-depth understanding of cross-sectoral collaboration mechanisms and policy-level challenges. Future research should include a broader range of policy makers and implementing entities.

## Experience and Lessons

The integrated eldercare-healthcare model demonstrates the potential of multi-level resource coordination but faces systemic challenges. Key successes include streamlined patient transfers through joint assessments, leveraging community-based services like daycare respite and rehabilitation beds to alleviate family burdens. However, structural contradictions emerge, such as Medicare restrictions on bed turnover and mismatched demand-capacity ratios, leading to operational bottlenecks (e.g., rehabilitation beds overwhelmed by unqualified referrals). Communication gaps—both physical (e.g., outreach failing bedridden seniors) and cognitive (e.g., misunderstood service terms)—hinder accessibility, exacerbated by poor digital and community synergy. Collaborative tensions arise from temporal conflicts (e.g., discharge delays) and ambiguous accountability between hospitals, nursing homes, and agencies, often rooted in institutional misalignment (e.g., Medicare audits vs. eldercare risks). Lessons highlight the need for clearer referral protocols, enhanced cross-sector coordination, and culturally tailored outreach to bridge service gaps while addressing regulatory and resource constraints. These findings may also apply to other Chinese cities and middle-income countries facing similar resource constraints and fragmented systems. Institutional barriers, weak referral mechanisms, and limited community care capacity are common challenges. Thus, lessons from Shanghai’s “Six-Bed Integration” pilot can inform broader reforms to strengthen coordination, accountability, and sustainability in integrated eldercare.

## Conclusion

This study empirically analyzes the “Six-Bed Integration” model in Xuhui District, Shanghai, uncovering core tensions and optimization pathways in implementing integrated care systems in China’s super-aged urban communities. The model connects three types of medical and eldercare beds to enable cross-setting service continuity, yet faces three major barriers: conflict between cost control and continuity of care, unclear referral responsibilities leading to risk avoidance, and digital divides disrupting service accessibility. These reflect systemic challenges in integrated care. Coordinated short-term policy adjustments and long-term institutional reforms are recommended, including the development of age-friendly digital tools. The Xuhui case highlights the need to balance cost, quality, and equity in China’s integrated care reform, offering insights for aging cities worldwide.

## Data Availability

The datasets generated and analyzed during this study (e.g., interview transcripts) are not publicly available due to ethical restrictions and participant confidentiality agreements. Anonymized excerpts of the data are available from the corresponding author upon reasonable request, subject to approval by the institutional ethics committee.
